# The role and potential mechanism of p75NTR in mineralization via in vivo p75NTR knockout mice and in vitro ectomesenchymal stem cells

**DOI:** 10.1111/cpr.12758

**Published:** 2020-01-10

**Authors:** Manzhu Zhao, Yingying Wang, Gang Li, Jun Li, Kun Yang, Chang Liu, Xiujie Wen, Jinlin Song

**Affiliations:** ^1^ College of Stomatology Chongqing Key Laboratory for Oral Diseases and Biomedical Sciences Chongqing Municipal Key Laboratory of Oral Biomedical Engineering of Higher Education Chongqing Medical University Chongqing China; ^2^ Department of Stomatology Daping Hospital & Research Institute of Surgery Third Military Medical University Chongqing China; ^3^ Department of Orthodontics Hospital of Stomatology Southwest Medical University Luzhou China

**Keywords:** ectomesenchymal stem cells, knockout mice, mineralization, p75 neurotrophin receptor, tooth

## Abstract

**Objective:**

The aim of this study is to investigate the role and potential mechanism of p75NTR in mineralization in vivo using p75NTR‐knockout mice and in vitro using ectomesenchymal stem cells (EMSCs).

**Materials and methods:**

Femur bone mass and daily incisor mineralization speed were assessed in an in vivo p75NTR‐knockout mouse model. The molecular signatures alkaline phosphatase (ALP), collagen type 1 (Col1), melanoma‐associated antigen (Mage)‐D1, bone sialoprotein (BSP), osteocalcin (OCN), osteopontin (OPN), distal‐less homeobox 1 (Dlx1) and Msh homeobox 1 (Msx1) were examined in vitro in EMSCs isolated from p75NTR^+/+^ and p75NTR^ExIII−/−^ mice.

**Results:**

p75NTR‐knockout mice were smaller in body size than heterozygous and wild‐type mice. Micro‐computed tomography and structural quantification showed that the osteogenic ability of p75NTR^ExIII^‐knockout mice was significantly decreased compared with that of wild‐type mice (*P* < .05). Weaker ALP and alizarin red staining and reduced expression of ALP, Col1, Runx2, BSP, OCN and OPN were also observed in p75NTR^ExIII−/−^ EMSCs. Moreover, the distance between calcein fluorescence bands in p75NTR^ExIII^‐knockout mice was significantly smaller than that in wild type and heterozygous mice (*P* < .05), indicating the lower daily mineralization speed of incisors in p75NTR^ExIII^‐knockout mice. Further investigation revealed a positive correlation between p75NTR and Mage‐D1, Dlx1, and Msx1.

**Conclusion:**

p75NTR not only promotes osteogenic differentiation and tissue mineralization, but also shows a possible relationship with the circadian rhythm of dental hard tissue formation.

## 1 INTRODUCTION

The p75 neurotrophin receptor (p75NTR) is a 75‐kDa transmembrane protein that is a member of the tumour necrosis factor receptor (TNFR) superfamily and is also known as nerve growth factor receptor, TNFR superfamily member 16 or CD271.^1^ It has been reported to participate in multiple intracellular signalling pathways to regulate a wide range of biological functions, including stem cell differentiation, cell adhesion, tumour cell invasion, apoptosis, signal transduction and metastasis.^2^, ^3^, ^4^, ^5^ p75NTR has been widely used as a marker of isolated oesophageal epithelial stem cells,^6^ adipose tissue‐derived mesenchymal stem cells (MSCs),^7^ and MSCs in the growth zones of regenerating fallow deer antlers and from the pedicle periosteum.^8^

Recently, reports have increasingly shown that p75NTR is involved in tooth morphogenesis and development.^9^, ^10^ p75NTR has been used as a marker of isolated cranial neural crest‐derived ectomesenchymal stem cells (EMSCs),^11^, ^12^ which present a useful stem cell model to investigate the mechanism of tooth morphogenesis and development. This is because this stem cell population gives rise to cells of the dental papilla and dental follicle, which subsequently form all tooth tissues except for enamel. In subsequent studies, p75NTR was reportedly positively related to the in vitro mineralization of EMSCs and promoted EMSCs differentiating into cells such as cementoblasts and odontoblasts.^13^, ^14^, ^15^ We previously revealed that p75NTR was strongly expressed at the cap and bell stages during tooth development and showed similar expression patterns as those of the mineralization‐related marker Runx2,^16^ implying the important role of p75NTR in tooth morphogenesis, especially during dental hard tissue mineralization. Some researchers have speculated that the effect of p75NTR on tooth development might be related to the Wnt/β‐catenin pathway and the factors sclerostin and melanoma‐associated antigen (Mage)‐D1.^13^, ^17^, ^18^ However, regulation by these transcription factors alone does not sufficiently explain the exact molecular mechanism of p75NTR in tooth morphogenesis and development.

p75NTR‐knockout mice are a good animal model used to investigate the specific role and mechanism of p75NTR in the morphogenesis of a variety of organs.^19^ There are two types of p75NTR‐knockout mice used in the literature, namely p75NTR^ExIII^‐ and p75NTR^ExIV^‐knockout mice.^20^, ^21^ p75NTR^ExIII^‐knockout mice are hypomorphic because they express a short variant of p75NTR as a consequence of alternative splicing. p75NTR^ExIV^‐knockout mice were created by deleting exon IV, resulting in a loss of both the full‐length and short isoform of p75NTR. p75NTR^ExIII^‐knockout mice, which were reported by Lee et al^22^ in 1992, were selected for this study. These mutant mice have a targeted deletion of exon III of p75NTR, and no functional p75NTR mRNA, protein or crosslinked products were detected in homozygous embryos. Genetic rescue further confirmed that the mutant phenotype described above was caused by the targeted mutation of the gene encoding p75NTR. Previous reports have shown that homozygous mice were a good model for studies on the biological functions of p75NTR.^20^, ^21^, ^22^, ^23^ p75NTR^ExIII^‐knockout mice, obtained from the Jackson Laboratory, were used to reveal the role and potential mechanism of p75NTR in mineralization during tooth morphogenesis and development.

Previous studies have indicated that p75NTR might play an important role in tooth morphogenesis and EMSC mineralization. However, the exact mechanism is unclear. The present study aimed to elucidate the role and potential mechanism of p75NTR in tooth morphogenesis and tissue mineralization via an in vivo study of p75NTR‐knockout mice and an in vitro study of EMSCs isolated from both p75NTR^ExIII−/−^ and p75NTR^+/+^ mice. The findings will contribute to the understanding of the molecular mechanism underlying tooth development and promote dental tissue engineering.

## 2 MATERIALS AND METHODS

### 2.1 Genotype identification of p75NTR‐knockout mice

**Figure 1:**
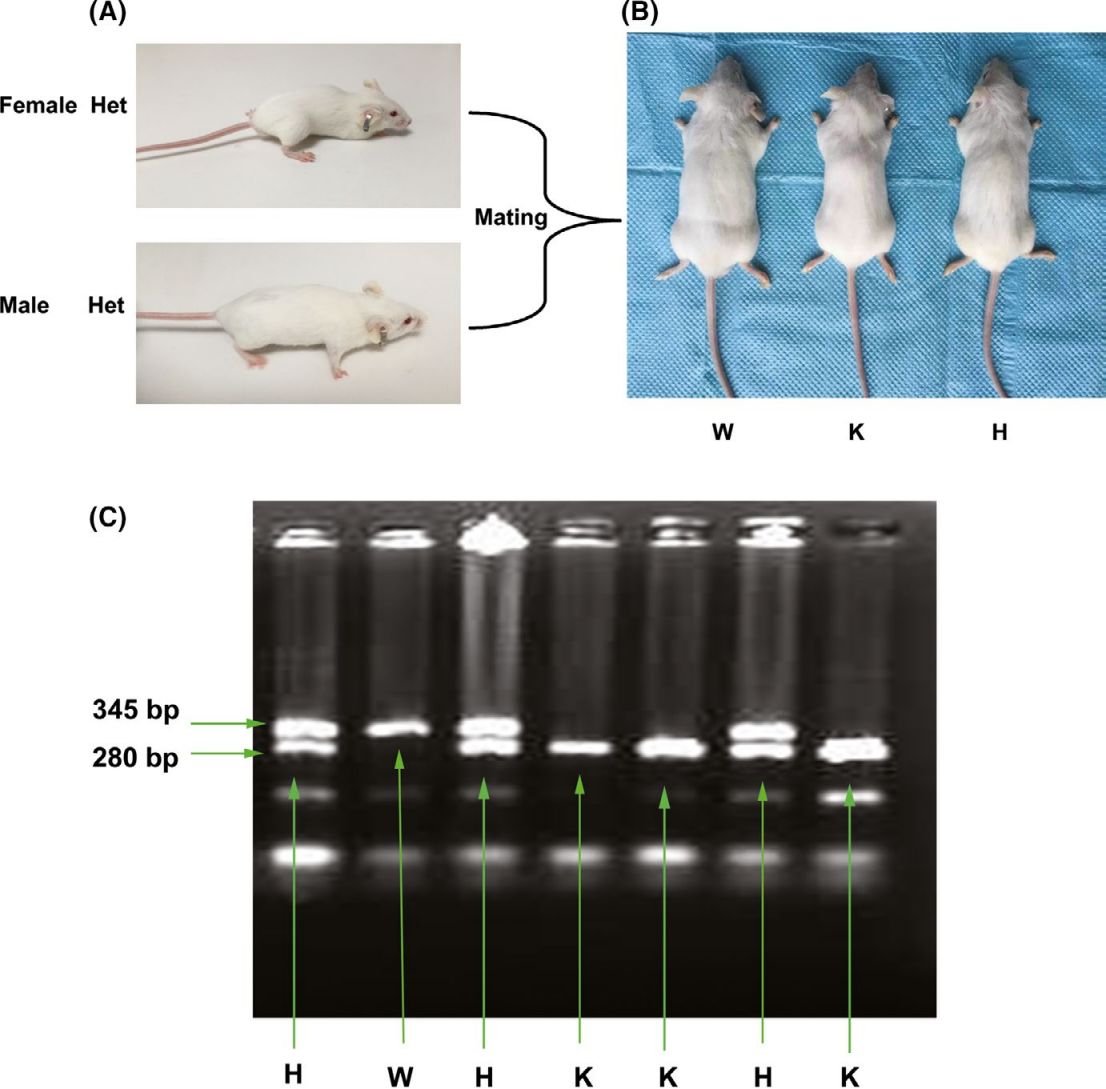
Generation and genotyping of p75NTR‐knockout mice. Heterozygous female and male (A) were mated to generate the three types of littermates: p75NTR‐knockout, wild type and heterozygous (B). The littermates with bands detected at both 280 bp and 345 bp were identified as heterozygous mice, and those with one band detected at 280 bp or 345 bp only were identified as p75NTR^ExIII−/−^‐knockout or wild‐type (p75NTR^+/+^) mice, respectively (C). Abbreviations: H, heterozygous; K, knockout; W, wild type

p75NTR‐knockout mice were obtained from the Jackson Laboratory and housed under specific‐pathogen‐free conditions (22°C, 12/12‐hour light/dark cycle, 50%‐55% humidity) in the Chongqing Medical University Animal Laboratory. All animal experiments were performed in accordance with protocols approved by the Medical Ethics Committee of the Chongqing Medical University. p75NTR‐knockout and wild‐type littermates were generated by mating between heterozygous females and males (Figure 1A and 1B). Genotyping of tail DNA was performed to distinguish p75NTR‐knockout from wild‐type and heterozygous progenies by polymerase chain reaction (PCR) as previously described^24^ (Figure 1B).

### 2.2 Calcein fluorescence assay

Nine newborn male mice (three for each group: p75NTR‐knockout, wild type and heterozygous) were routinely fed for 40 days. Calcein was administered by intraperitoneal injection at 25 mg/kg every fifth day for four times, and the mice were sacrificed at the second day after the final injection. The calcein fluorescence assay was performed as previously described.^25^ Lower jaws dissected from p75NTR‐knockout, wild‐type and heterozygous mice were fixed in 2.5% glutaraldehyde and dehydrated with ethanol in a graded concentration series. The hard tissue specimens were embedded, sliced at 8 μm with the EXAKT precision cutting and grinding system (EXAKT Vertriebs GmbH) and observed using an upright fluorescence microscope (Olympus) with excitation and emission wavelengths of 485 nm and 510 nm, respectively. The distance between the observed fluorescence bands was measured, and the mineral apposition rate (μm/d) was calculated using Image‐Pro Plus 6.0.

### 2.3 Micro‐computed tomography and microstructural quantification

Male wild‐type (p75NTR^+/+^) and p75NTR‐knockout (p75NTR^ExIII−/−^) mice (three in each group) were routinely fed and sacrificed at 8 weeks of age for micro‐computed tomography (micro‐CT) evaluation. Femurs were dissected, fixed in 4% polyoxymethylene and scanned with micro‐CT (viva ct40 Scanco Medical AG). The images were analysed using MicroView software version 2.2. The following microstructural parameters were quantitatively analysed: bone volume (BV), bone volume/total volume (BV/TV, bone volume fraction), bone surface/bone volume ratio (BS/BV), trabecular number (Tb.N), trabecular thickness (Tb.Th), trabecular separation (Tb.Sp), cortical bone volume (Ct.BV), cortical bone surface/bone volume ratio (Ct.BS/BV) and cortical thickness (Ct.Th).

### 2.4 Isolation and culture of p75NTR^+/+^ and p75NTR^ExIII−/−^ EMSCs

Eight embryos were obtained from heterozygous mice (Figure 4A) that were 13.5 days into embryonic development (E13.5). Each embryo was separately used for EMSC isolation (Figure 4B and 4C), as previously described,^25^ and genotype identification (Figure 4I). Embryonic maxillofacial processes were dissected, minced into fine pieces (Figure 4D‐4F), digested with 1% trypsin/1 mmol/L ethylenediaminetetraacetic acid (EDTA) solution (Sigma), filtered through a 75‐μm mesh filter, centrifuged and cultured in Dulbecco's Modified Eagle Medium/Ham's F12 (DMEM/F12) (Gibco) supplemented with 10% foetal bovine serum (FBS, Gibco) at 37°C in a humidified incubator containing 5% CO_2_. The p75NTR^+/+^ and p75NTR^ExIII−/−^ EMSCs were determined according to PCR genotyping as previously described.^24^

### 2.5 Identification of p75NTR^+/+^ and p75NTR^ExIII−/−^ EMSCs

Flow cytometry was carried out to identify isolated p75NTR^+/+^ and p75NTR^ExIII−/−^ EMSCs. Cells at passage 3 were harvested and fixed in 4% polyoxymethylene for 30 minutes followed by overnight incubation at 4°C with mouse anti‐mouse CD29, CD45, CD90 and CD146 primary antibodies (sc‐9970, sc‐53047, sc‐53116 and sc‐28667, respectively; 1:100; Santa Cruz Biotechnology) according to the manufacturer's protocol. The corresponding anti‐mouse IgG‐fluorescein isothiocyanate (FITC) (ab6785, 1:100, Abcam) and anti‐mouse IgM‐FITC (ab8517, 1:100, Abcam) secondary antibodies were added. The cells were analysed using a FACS Calibur flow cytometer (BD Biosciences).

### 2.6 Cell cycle assay

p75NTR^+/+^ and p75NTR^ExIII−/−^ EMSCs at passages 3 were collected for cell cycle analysis. The cells were trypsinized with 1% trypsin/1 mmol/L EDTA solution and centrifuged at 800 rpm for 5 minutes. The supernatant was removed, and the cells were washed twice with phosphate‐buffered saline (PBS). Then, 2 mL of cold 70% dehydrated alcohol was added quickly to fix the cells at 4°C for 24 hours. The samples were washed with PBS and incubated with 100 μg/mL RNase A at 4°C for 30 minutes. Thereafter, the cells were filtered, stained with 2 mg/mL propidium iodide at 4°C for 30 minutes and analysed using FACS Calibur flow cytometry.

### 2.7 Growth curve and cell counting kit‐8 assay

p75NTR^+/+^ and p75NTR^ExIII−/−^ EMSCs at passages 3 were seeded into 96‐well plates at a density of 1 × 10^3^ cells/well. The cells were counted every day and evaluated using the cell counting kit‐8 (CCK‐8) assay (Dojindo Kagaku Co) according to the manufacturer's instructions. Growth curves were generated for the two cell populations, and the population doubling times (PDT) were calculated using the formula PDT = T × log2/(logNt – logN0), where T represents day of culture, Nt represents the number of cells on day T, and N0 represents the number of cells on day 0. To determine the number of viable cells in each well, the absorbance of the plate was measured using a microplate reader at 450 nm. Cell proliferation was represented as the mean ± standard deviation of the absorbance of five wells.

### 2.8 Alkaline phosphatase and alizarin red staining

p75NTR^+/+^ and p75NTR^ExIII−/−^ EMSCs were seeded in 6‐well plates at a density of 1 × 10^5^ cells/well and incubated in osteogenic medium (containing 50 mg/mL ascorbic acid, 10 mmol/L β‐glycerol phosphate and 10 − 8 m dexamethasone), and the medium was changed every 3 days. On days 3 and 7, the cells were washed twice with PBS, fixed in 4% paraformaldehyde for 30 minutes and subjected to alkaline phosphatase (ALP) staining using a kit (Beyotime) according to the manufacturer's instructions. After 14 days of incubation in osteogenic medium, the cells were subjected to both ALP and alizarin red staining (Sangon). After staining, the cells were washed three times with distilled water and observed using a phase‐contrast microscope.

### 2.9 Real‐time PCR

Total RNA was extracted from approximately 1 × 10^6^ EMSCs using Trizol reagent (Invitrogen) according to the manufacturer's protocol. RNA was quantified and reverse‐transcribed into cDNA using the RevertAidTM First Strand cDNA Synthesis Kit (MBI Fermentas) according to the manufacturer's instructions. Real‐time PCR (RT‐PCR) was performed as previously described^12^ to further confirm the findings on the role and mechanism of p75NTR in mineralization. PCR amplification was performed for 30 cycles in a thermal cycler, with initial denaturation at 94°C for 30 seconds, subsequent annealing at 60°C for 60 seconds and extension at 72°C for 90 seconds. The PCR products were visualized on a 1.5% agarose gel containing 5 mg/mL ethidium bromide. The primers used are listed in Table 1.

**Table 1:** Specific primers used for RT‐PCR

Gene	Primer sequences	GenBank^®^ Accession no.
OPN	Forward: 5′‐TTCTCCTGGCTGAATTCTGAGG‐3′ Reverse: 5′‐GCTATAGGATCTGGGTGCAGG‐3′	NM_001204203
OCN	Forward: 5′‐CTTGGTGCACACCTAGCAGA‐3′ Reverse: 5′‐GCCGGAGTCTGTTCACTACC‐3′	X04142
BSP	Forward: 5′‐GGCGACACTTACCGAGCTTA‐3′ Reverse: 5′‐GGGGGCTTCACTGATGGTAG‐3′	NM_008318
ALP	Forward: 5′‐GGCTCTGCCGTTGTTTCTCT‐3′ Reverse: 5′‐AAGGTGCTTTGGGAATCTGC‐3′	NM_007431
ColⅠ	Forward: 5′‐GGTCCTTCTGGTCCTCGTG‐3′ Reverse: 5′‐TCTCCGTTCTTGCCAGGA‐3′	NM_007732
Runx2	Forward: 5′‐CTGCCACCTCTGACTTCTGC‐3′ Reverse: 5′‐GATGAAATGCCTGGGAACTG‐3′	NM_001146038
GAPDH	Forward: 5′‐ACAGCAACAGGGTGGTGGAC‐3′ Reverse: 5′‐TTTGAGGGTGCAGCGAACTT‐3′	NM_008084

### 2.10 Western blot

Total proteins were extracted from approximately 1 × 10^6^ EMSCs using radioimmunoprecipitation assay buffer (Beyotime), and the protein concentrations were determined using a bicinchoninic acid assay (Beyotime). The proteins (40 μg/lane) were separated by 10% sodium dodecyl sulphate‐polyacrylamide gel electrophoresis and then transferred to polyvinylidene fluoride membranes (Millipore). After blocking with 5% bovine serum albumin for 2 hours at room temperature, the membranes were incubated overnight at 4°C with the following primary antibodies: rabbit polyclonal GAPDH (AP0063, 1:2000; Immunoway), rabbit polyclonal Runx2 (ab23981, 1:1000; Abcam), mouse monoclonal collagen type 1 (Col‐1, ab6308, 1:1500; Abcam), mouse monoclonal ALP (ab95462, 1:1500; Abcam), mouse monoclonal bone sialoprotein (BSP, ab125227, 1:1500; Abcam), mouse monoclonal osteocalcin (OCN, ab93876, 1:1500; Abcam), mouse monoclonal osteopontin (OPN, sc‐21742, 1:1500; Santa Cruz Biotechnology), rabbit polyclonal Mage‐D1 (sc‐28243, 1:500; Santa Cruz Biotechnology), rabbit polyclonal distal‐less homeobox 1 (Dlx1, sc‐81959, 1:500; Santa Cruz Biotechnology) or rabbit polyclonal Msh homeobox 1 (Msx1, ab174207, 1:500; Abcam). Then, the membranes were washed and incubated with horseradish peroxidase‐conjugated anti‐mouse or anti‐rabbit secondary antibodies. Greyscale analysis for all blots was performed with Quantity One software (Bio‐Rad). Relative protein expression was determined by the ratio of the greyscale value of the target protein to that of the internal reference GAPDH.

### 2.11 Immunohistochemical staining

Embryonic maxillofacial processes were dissected from mice at E13.5 d, E15.5 d and E18.5 d. The tissues were fixed in 4% paraformaldehyde and sectioned at 6 μm for immunostaining. The following primary antibodies were used: rabbit anti‐mouse p75NTR (ab8847, 1:1500; Abcam) and rabbit anti‐mouse Mage‐D1 (sc‐28243, 1:1500; Santa Cruz Biotechnology). Then, the specimens were treated with the DAB Detection Kit Streptavidin‐Biotin (ZSGB) according to the manufacturer's protocols, followed by visualization under a phase‐contrast microscope.

### 2.12 Mage‐D1 siRNA transfection in p75NTR^+/+^ EMSCs

p75NTR^+/+^ EMSCs at passage 3 were seeded in a 6‐well plate until the cells reached 70%‐90% confluence after 24 hours of incubation. The culture medium was changed to DMEM/F12 (without penicillin, streptomycin and FBS) 2 hours before transfection. We transfected p75NTR^+/+^ EMSCs with siRNA targeting Mage‐D1 (sense, 5′‐GCUUGGAAUGACACCACUATT‐3′, antisense, 5′‐UAGUGGUGUCAUUCCAAGCTT‐3′) or negative control siRNA (sense, 5′‐AUAGCCGUACCAUAAGUGCTT‐3′, antisense, 5′‐GCACUUAUGGUACGGCUAUTT‐3′) that has been tested for the absence of specific degradation of any known cellular mRNA (GenePharma). The siRNA (20 pmol) and Lipofectamine 2000 (1 μL) were separately dissolved in 50 μL of Opti‐MEM (Invitrogen). After 5 minutes of shaking, the two solutions were mixed, and 100 μL of the siRNA/Lipofectamine 2000 mixture was added to the p75NTR^+/+^ EMSCs. After 24 hours of incubation, the cells were harvested for Western blot.

### 2.13 Statistical analysis

Data for the calcein fluorescence assay, bone analysis, growth curve and CCK‐8 assays, greyscale analysis and RT‐PCR were presented as the mean ± standard deviation. Statistical significance was assessed using Prism 5 (GraphPad Software). Comparisons were made using a *t* test or one‐way analysis of variance (Tukey's test) for experiments involving more than three groups. All experiments were performed three times, and differences were considered significant at *P* < .05.

## 3 RESULTS

### 3.1 Identification and visual observation of p75NTR‐knockout mice

The genotyping results are shown in Figure 1C. The littermates with two bands detected at 280 bp and 345 bp were identified as heterozygous mice, and those with one band detected at either 280 bp or 345 bp only were identified as p75NTR^ExIII−/−^‐ knockout or wild‐type (p75NTR^+/+^) mice, respectively. When the littermates grew to 8 weeks of age, an obvious difference in body size was observed (Figure 1B). p75NTR‐knockout mice (length 7.2 cm; weight 18.6 g) were smaller than the wild‐type (length 8.7 cm; weight 21.1 g) and heterozygous mice (length 8.4 cm; weight 20.6 g).

### 3.2 Daily incisor mineralization speed

**Figure 2:**
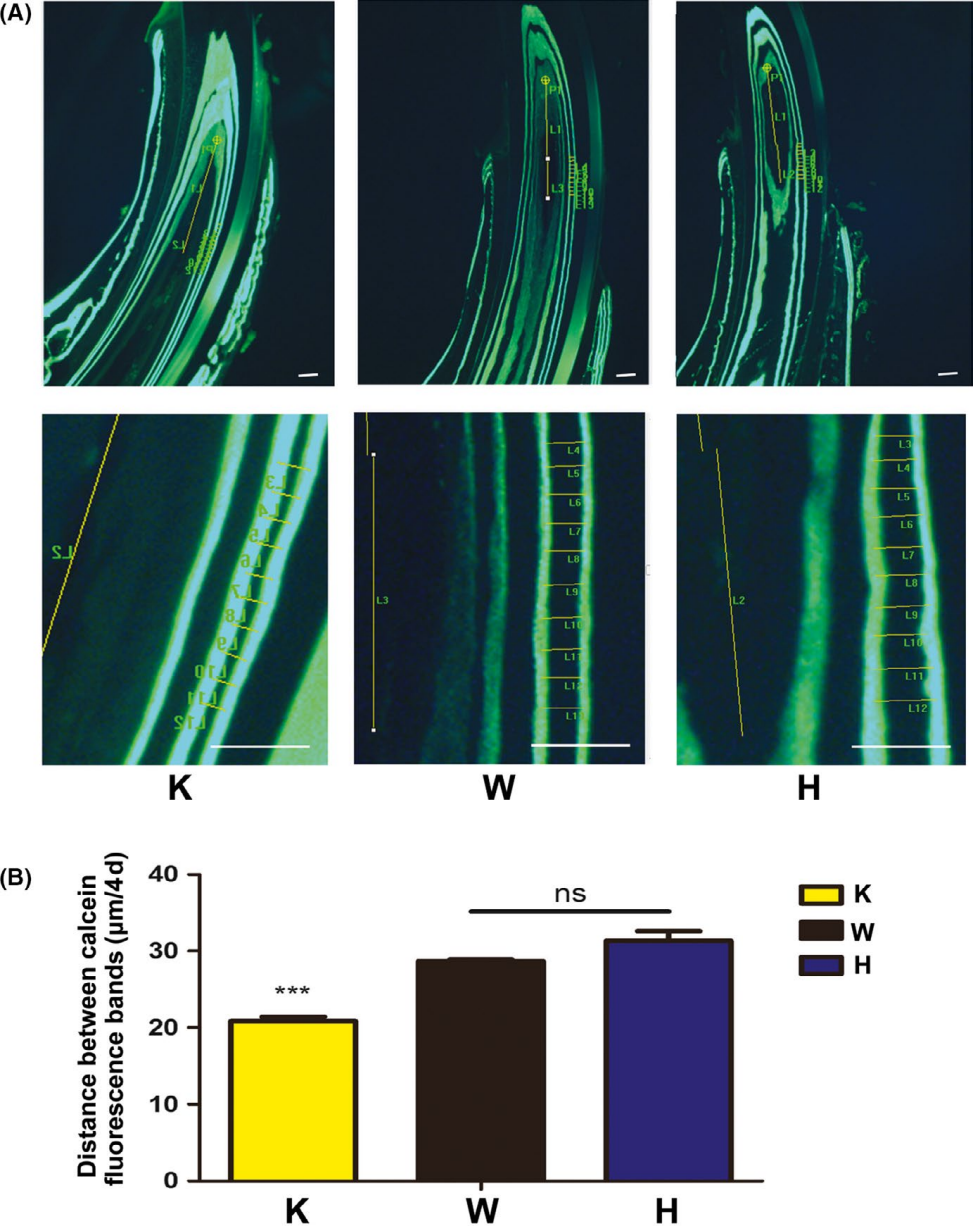
Results of calcein fluorescence assay. Fluorescence microscopic observation showed that the distance between the calcein fluorescence bands in p75NTR‐knockout mice was distinctly shorter than that in wild‐type and heterozygous mice (A).The distance of every fifth day in p75NTR‐knockout mice was 20.84 μm, which was significantly lower than that of 28.72 μm in wild‐type and 31.60 μm in heterozygous mice (*P* < .01) (B). No significance was found between wild‐type and heterozygous mice (*P* > .05). Scale bar represents 50 μm. Abbreviations: H, heterozygous; K, knockout; W, wild type

Fluorescence microscopic observation showed that the daily mineralization speeds of the incisors were different between the three types of mice, as observed in the calcein fluorescence assay (Figure 2A). The distance between the calcein fluorescence bands, representing the mineralization on every fifth day, was 20.84 μm in p75NTR‐knockout mice, which was significantly lower than that in wild‐type (28.72 μm) and heterozygous mice (31.60 μm) (Figure 2B; *P* < .01). No significance was found between wild‐type mice and heterozygous mice (*P* > .05). The data indicated that p75NTR might participate in the regulation of the daily mineralization speed of mouse incisors.

### 3.3 Bone mass of p75NTR‐knockout and wild‐type mice

**Figure 3:**
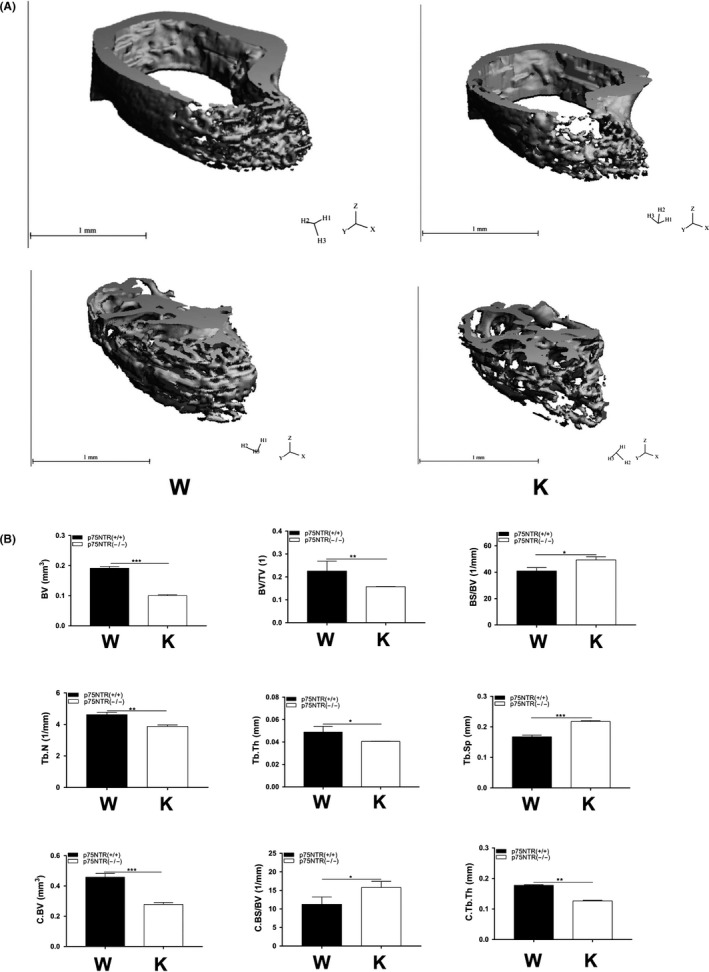
Micro‐computed tomography observation and structural parameter quantification. The size and thickness of cortical bone, as well the size and density of trabecular bone in p75NTR‐knockout mice were significantly smaller than those in wild‐type mice (A). Quantification of the structural parameters (B) showed that BV, BV/TV, Tb.N, Tb.Th, Ct.BV and Ct.Th were significantly lower in the p75NTR‐knockout mice compared with those in wild‐type mice (*P* < .05), and conversely, BS/BV, Ct.BS/BV and Tb.Sp were significantly higher (*P* < .05). Abbreviations: K, knockout; W, wild type

Micro‐CT observations revealed obvious bone loss in both the femur trabecular and cortical bone of p75NTR‐knockout mice (Figure 3A). The size and thickness of the cortical bone in the p75NTR‐knockout mice were significantly reduced compared with those in the wild‐type mice, as were the size and density of trabecular bone. Quantification of the structural parameters (Figure 3B) indicated that the BV, BV/TV, Tb.N, Tb.Th, Ct.BV and Ct.Th of the p75NTR‐knockout mice were significantly lower than those of the wild‐type mice (*P* < .05), implying smaller bone size and thickness. In contrast, the BS/BV, Ct.BS/BV and Tb.Sp of the p75NTR‐knockout mice were significantly higher than those of the wild‐type mice (*P* < .05), signifying lower bone density. The results of both micro‐CT and quantification analysis showed that osteogenic potential was evidently impaired when p75NTR was deleted.

### 3.4 Phenotype and proliferation of p75NTR^+/+^ and p75NTR^ExIII−/−^ EMSCs

**Figure 4:**
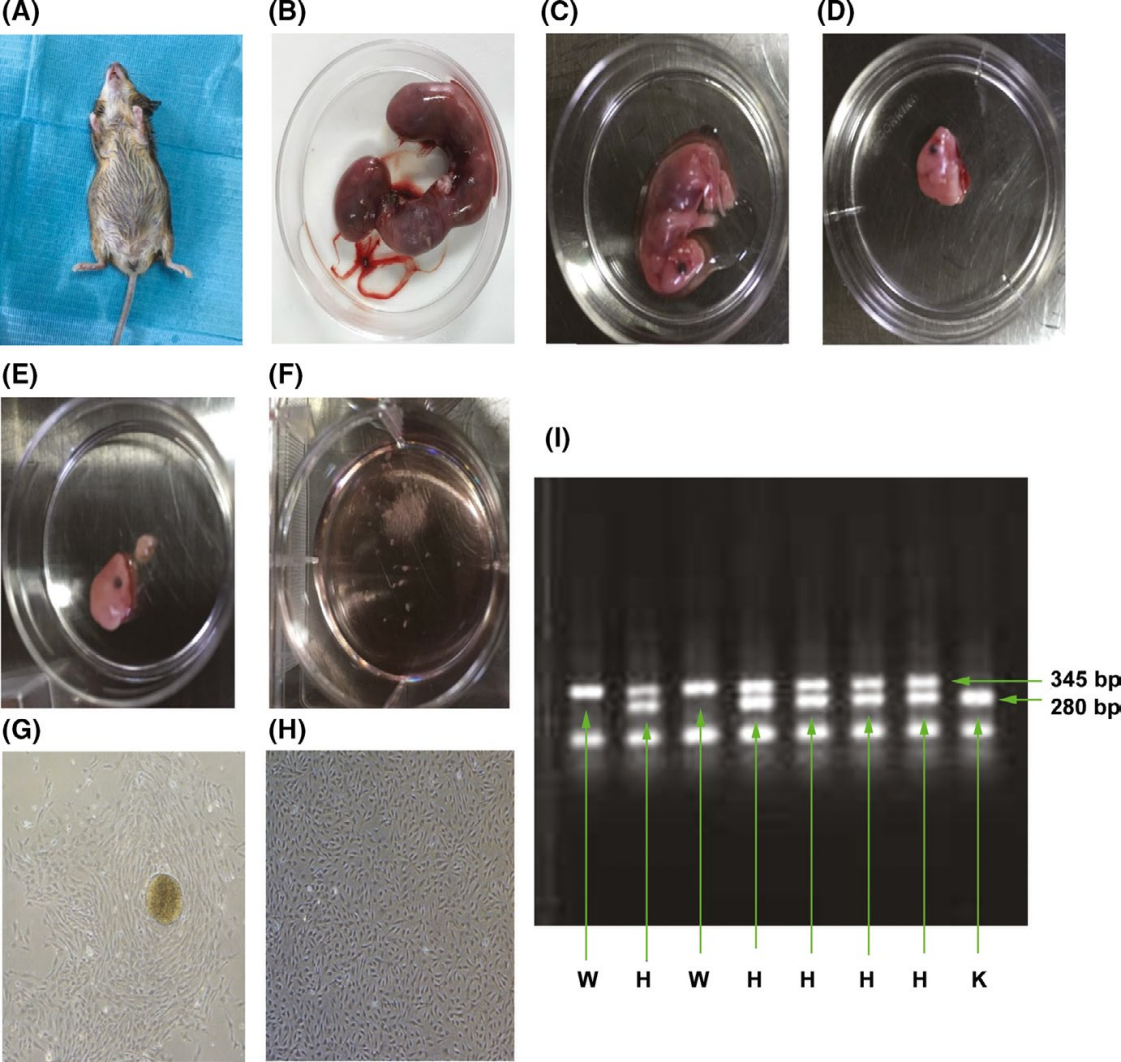
Isolation and genotypic identification of mouse embryonic EMSCs. E13.5 heterozygous mice were selected and each embryo was separated (A, B). The embryonic maxillofacial processes were dissected and minced into fine pieces for the culture of EMSCs (C‐F). EMSCs exhibited a fibroblast‐like morphology (G, H). Genotypic identification was shown for each embryo used to isolate EMSCs (I). Scale bar represents 50 μm. Abbreviations: H, heterozygous; K, knockout; W, wild type

**Figure 5:**
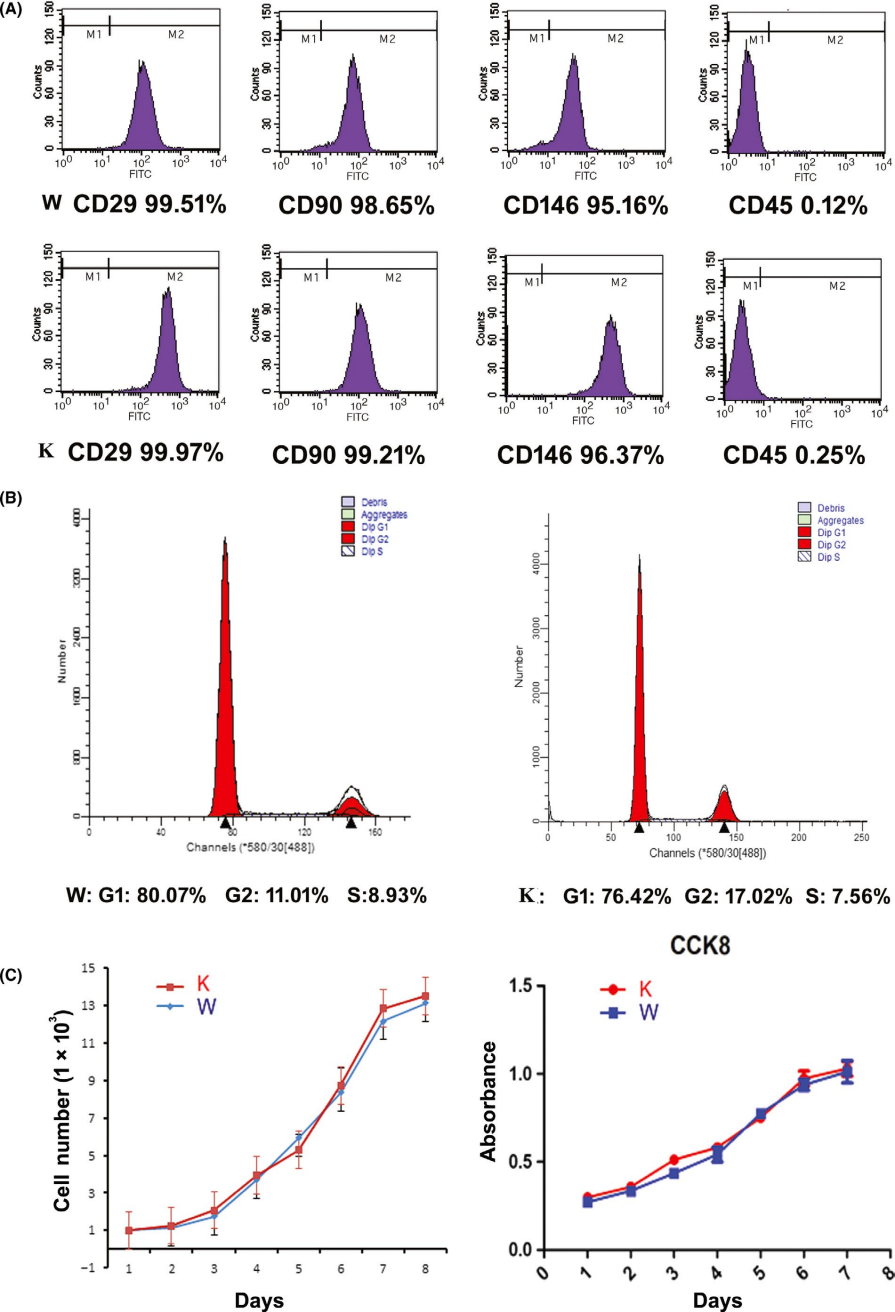
Characterization of p75NTR^+/+^ and p75NTR^ExIII−/−^ EMSCs. The MSC markers CD29, CD90 and CD146 were highly expressed in both p75NTR^ExIII−/−^ and p75NTR^+/+^ EMSCs, while the hematopoietic marker CD45 was hardly detected (A). Cell cycle assay showed no significant difference between p75NTR^+/+^ and p75NTR^ExIII−/−^ EMSCs in proliferation ability (B). Growth curves and CCK‐8 assay (C) showed that both cell populations began to grow exponentially on day 2 and the population doubling times were 29.17 h for p75NTR^ExIII−/−^ EMSCs and 28.92 h for p75NTR^+/+^ EMSCs, calculated by the formula PDT = T × log2/ (logNt – logN0), T: day of culture, Nt: number of cells on day T, N0: number of cells on day 0. Abbreviations: K, knockout; W, wild type

EMSCs exhibited a fibroblast‐like morphology (Figure 4G and 4H). Phenotypic analysis by flow cytometry showed that the MSC markers CD29, CD90 and CD146 were highly expressed in both p75NTR^ExIII−/−^ and p75NTR^+/+^ EMSCs while the hematopoietic marker CD45 was hardly detected, indicating that the isolated p75NTR^ExIII−/−^ and p75NTR^+/+^ EMSCs were characteristic of MSCs (Figure 5A). The proliferation assays showed that both cell populations began to grow exponentially from day 2 and the population doubling times were 29.17 hours for p75NTR^ExIII−/−^ EMSCs and 28.92 hours for p75NTR^+/+^ EMSCs. There was no significant difference in cell proliferation ability between p75NTR^+/+^ and p75NTR^ExIII−/−^ EMSCs in the cell cycle, growth curve and CCK‐8 assays (*P* > .05) (Figure 5B and 5C).

### 3.5 Mineralization ability of p75NTR^+/+^ and p75NTR^ExIII−/−^ EMSCs

**Figure 6:**
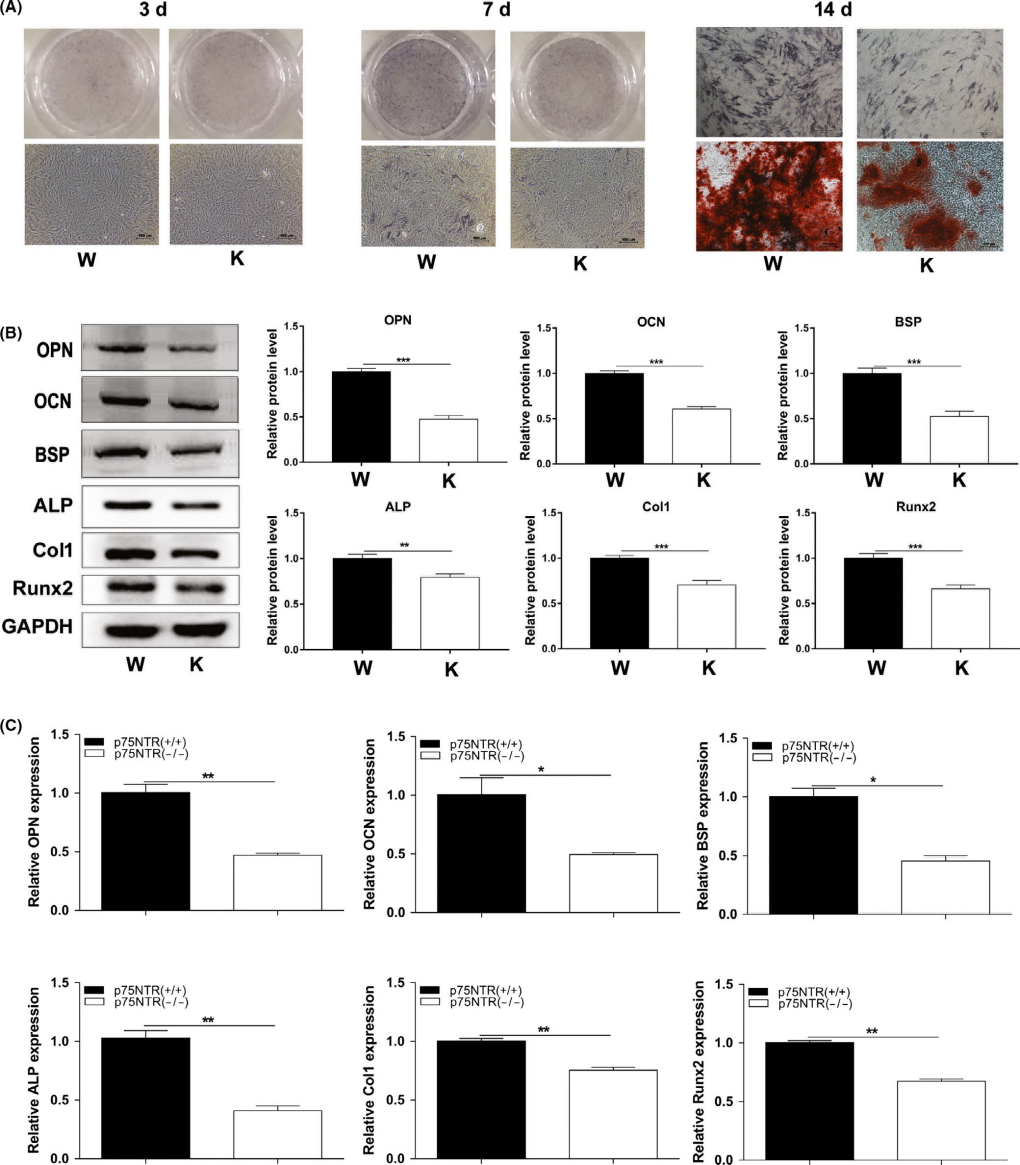
Mineralization assay. After three days of osteogenic induction, ALP staining was hardly detected in both p75NTR^+/+^ and p75NTR^ExIII−/−^ EMSCs (A), but on day 7, more abundant and deeper ALP staining was present in p75NTR^+/+^ EMSCs compared with that in p75NTR^ExIII−/−^ EMSCs. On day 14, this difference became more prominent in not only ALP but also alizarin red staining. ALP staining was deeper and the mineralized nodules of alizarin red staining were larger in p75NTR^+/+^ EMSCs. Western blot on day 14 showed increased expression of the mineralization‐related markers ALP, Col1, Runx2, BSP, OCN and OPN in p75NTR^+/+^ EMSCs (B). Similar results were obtained by RT‐PCR on day 14 (C). The mRNA expression of ALP, Col‐1, Runx2, BSP, OCN and OPN in p75NTR^+/+^ EMSCs was significantly higher than that in p75NTR^ExIII−/−^ EMSCs. Scale bar represents 100 μm. Abbreviations: K, knockout (p75NTR^ExIII−/−^); W, wild type (p75NTR^+/+^)

ALP staining was not initially detected in both p75NTR^+/+^ and p75NTR^ExIII−/−^ EMSCs after three days of osteogenic induction (Figure 6A), but staining was observed on day 7. Moreover, p75NTR^+/+^ EMSCs showed more abundant and deeper ALP staining compared with that in p75NTR^ExIII−/−^ EMSCs, and this difference became more prominent by day 14. Alizarin red staining also exhibited similar results, as more and larger mineralized nodules were found in p75NTR^+/+^ EMSCs on days 14. To confirm this difference, the mineralization‐related factors ALP, Col1, Runx2, BSP, OCN and OPN were detected by Western blot (Figure 6B) and RT‐PCR (Figure 6C). All of these factors were significantly higher in p75NTR^+/+^ EMSCs at both mRNA and protein levels (*P* < .05) after 14 days of culture in osteogenic medium. These results confirmed that p75NTR^ExIII−/−^ EMSCs possessed lower mineralization ability than p75NTR^+/+^ EMSCs.

### 3.6 Potential mechanism of p75NTR in regulating mineralization

**Figure 7:**
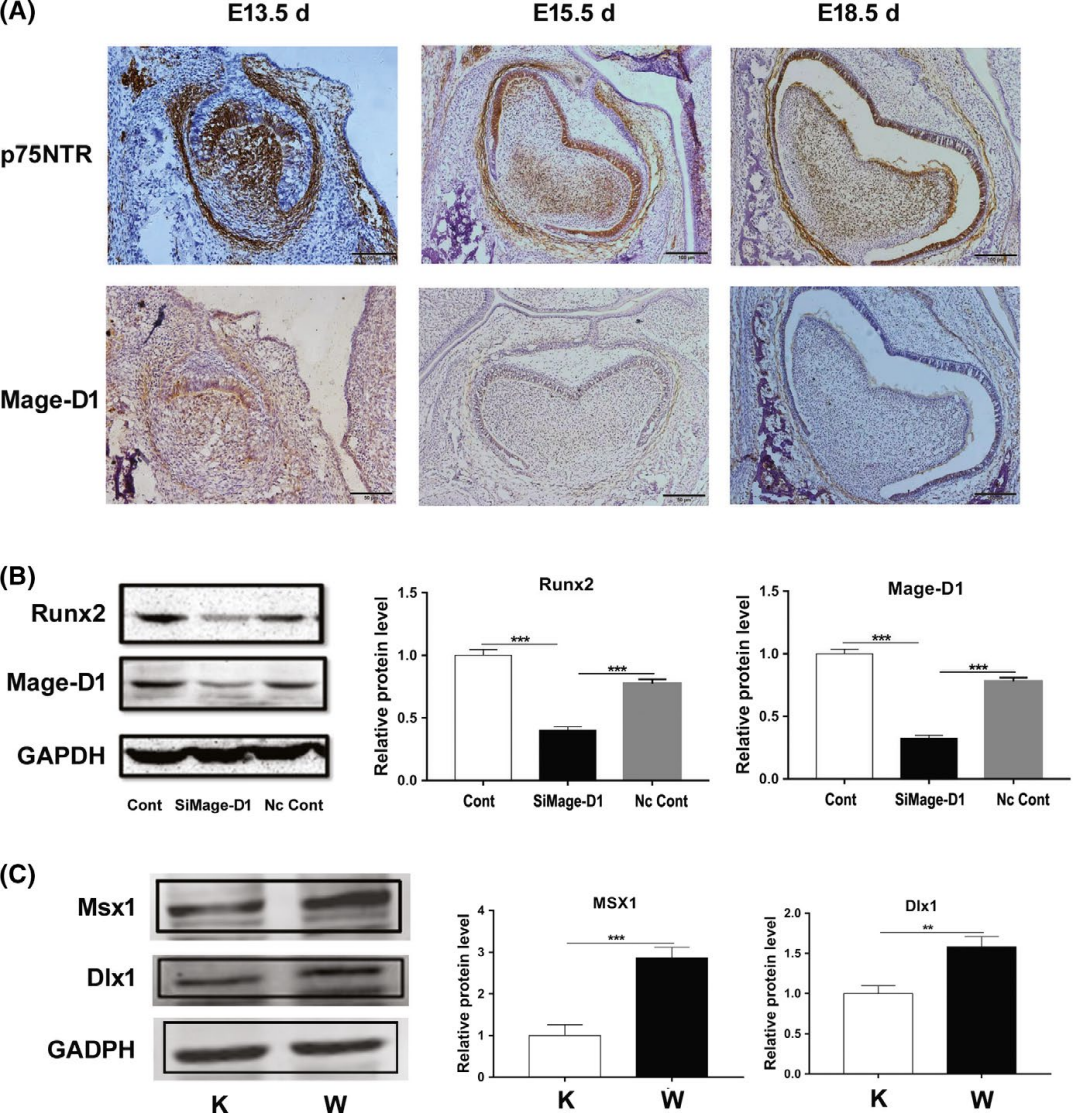
Investigations revealing the potential mechanism of p75NTR in mineralization. Immunohistochemistry showed similar expression patterns for p75NTR and Mage‐D1 (A). They were strongly expressed in the same areas at the cap stage (E13.5 d), but their expression became weak at the bell stage (E15.5 d) and at the beginning of dental hard tissue formation (E18.5 d). Western blot showed that Runx2 was significantly decreased when Mage‐D1 was down‐regulated by siRNA transfection (B). The homeobox genes Dlx1 and Msx1 were both weakly expressed in p75NTR^ExIII−/−^ EMSCs but strongly expressed in p75NTR^+/+^ EMSCs (C). Abbreviations: K, knockout; W, wild type

Based on a previous report,^16^ this study continued to investigate the potential mechanism of p75NTR in regulating mineralization. Immunohistochemical staining demonstrated that the expression patterns of p75NTR and Mage‐D1 were similar (Figure 7A), as they were strongly expressed in the mesenchyme of the dental papilla and dental follicle at the cap stage (E13.5 d). Their expressions then became slightly weaker in the mesenchyme but began to appear in the inner enamel epithelium at the bell stage (E15.5 d) and at the beginning of dental hard tissue formation (E18.5 d), signifying the exchange of odontogenic signals between the dental epithelium and the dental mesenchyme during tooth morphogenesis. Moreover, this similar pattern indicated that there may be a positive correlation between p75NTR and Mage‐D1 in mineralization during tooth development. Western blot after Mage‐D1 siRNA transfection confirmed this speculation. Runx2, a mineralization‐related marker, was significantly down‐regulated when Mage‐D1 was suppressed by siRNA treatment (Figure 7B), demonstrating that Mage‐D1 positively regulated Runx2 expression. To further examine the potential mechanism of p75NTR in tooth morphogenesis, the homeobox genes Dlx1 and Msx1 were detected. The results in Figure 7C indicate that both Dlx1 and Msx1 were weakly expressed in p75NTR^ExIII−/−^ EMSCs but strongly expressed in p75NTR^+/+^ EMSCs, revealing their positive correlation with p75NTR.

## 4 DISCUSSION

The sequential and reciprocal interactions between the oral epithelium and cranial neural crest‐derived mesenchyme trigger tooth morphogenesis.^26^, ^27^ In this process, EMSCs, which arise from the cranial neural crest, migrate to and populate the branchial arches, giving rise to cells of the dental papilla and dental follicle and subsequently forming dentin, pulp, cementum and periodontal ligaments.^28^ p75NTR, which is abundantly expressed in cranial neural crests, is considered a typical marker for this stem cell population.^29^ It has been used to successfully select cranial neural crest‐derived EMSCs (p75^+^ EMSCs) in vitro, providing a useful stem cell model for research on tooth morphogenesis.^12^ Moreover, p75NTR has been reported to participate in tooth morphogenesis and dental hard tissue mineralization.^13^, ^14^, ^15^, ^16^, ^17^ However, most molecular signatures on the effect of p75NTR in tooth morphogenesis remain to be uncovered, and the elusive mechanism underlying tooth development has severely restricted dental tissue engineering thus far. In this study, p75NTR^ExIII^‐knockout mice and EMSCs isolated from p75NTR^ExIII−/−^ and p75NTR^+/+^ male mice were investigated to reveal the exact mechanism of p75NTR in mineralization regulation, contributing to dental tissue engineering.

Knockout mice are a useful method of investigating the effects of a specific molecule and examining the potential mechanism within molecular signature networks. Recently, p75NTR‐knockout mice have been widely used in studies on neurological diseases and neural regeneration.^19^, ^30^, ^31^ This model greatly contributes to the understanding of disease pathogenesis and exploration of methods to reverse neurodegeneration or achieve nerve regeneration. In this study, p75NTR^ExIII^‐knockout mice were used to reveal the mechanism of tooth morphogenesis and tissue mineralization. Interestingly, p75NTR‐knockout mice were found to be smaller in body shape than wild‐type and heterozygous mice. Moreover, micro‐CT and bone microstructural parameters analysis in p75NTR‐knockout mice showed obvious bone loss in both the femur trabecular and cortical bone, implying that the osteogenic potential was remarkably decreased in the absence of p75NTR. These in vivo data confirmed the findings of previous in vitro studies on the effect of p75NTR in mineralization.^13^, ^17^, ^18^ Mineralization‐related markers such as Runx2, ALP, Col1, BSP, OCN and OPN have been reported to display a positive correlation with p75NTR in rat EMSCs in vitro. These findings supported the speculation that p75NTR plays an up‐regulatory role in the osteogenic differentiation of stem cells and hard tissue formation.

In previous studies on the effect of p75NTR in tooth development, EMSCs were mostly isolated from the facial processes of rat embryos. To confirm the abovementioned speculation, EMSCs were isolated from mice in this study. Both p75NTR^+/+^ and p75NTR^ExIII−/−^ EMSCs showed high proliferation ability and were well characterized as MSCs. However, a significant difference in cell mineralization ability was observed between the two stem cell populations. ALP and alizarin red staining were weak in p75NTR^ExIII−/−^ EMSCs, and the mineralization‐related genes ALP, Col1, Runx2, BSP, OCN and OPN were also expressed at low levels. These findings demonstrated that p75NTR^ExIII−/−^ EMSCs possessed lower osteogenic differentiation potential than p75NTR^+/+^ EMSCs, implying that the expression of p75NTR could contribute to the osteogenic differentiation of mouse stem cells. In addition, p75NTR participated in the regulation of mouse incisor mineralization. The daily incisor mineralization speed, which was evaluated by the distance between the calcein fluorescence bands, was significantly lower in p75NTR‐knockout mice than that in wild‐type and heterozygous mice. This finding indicated that p75NTR not only regulated cell differentiation and tissue mineralization, but also influenced the circadian rhythm of dental hard tissue formation, consistent with a report by Baeza‐Raja et al^32^ p75NTR was identified as a novel CLOCK‐controlled gene because alterations in the rhythmic expression of p75NTR have been detected in the suprachiasmatic nucleus and liver using CLOCK^ExIII−/−^ and CLOCK^Δ^ 19 mice. Non‐canonical E‐boxes, which are a DNA sequence with protein binding sites and could be directly bound by CLOCK/BMAL1, were also reported in p75NTR. CLOCK and BMAL1 are known to regulate the gene expression of CLOCK by interacting with a promoter element termed the E‐box (CACGTG).^33^

It is well known that the formation of dental hard tissues is periodic, and the growth lines are an evidence of this phenomenon. Based on p75NTR‐knockout mice, we found that p75NTR might play a potential role in the circadian rhythm and the space between the growth lines during tooth development. To further elucidate the mechanism, Mage‐D1, a member of the MAGE gene family, was detected. Mage‐D1 was first found to be expressed in multiple tissues by Põld et al^34^ in 1999 and have more complex functions than other members of the MAGE gene family. Yang et al^13^ reported that Mage‐D1 might be a possible downstream factor of p75NTR in the regulation of rat EMSC mineralization. Our immunohistochemistry results showed that Mage‐D1 was strongly expressed in the dental follicle, dental papilla, odontoblastic layer and inner enamel epithelium, similar to the expression pattern of p75NTR. Moreover, subsequent siRNA intervention revealed a positive correlation between p75NTR and Mage‐D1 in osteogenic differentiation. Our data confirmed the above speculation that Mage‐D1 might be a downstream factor of p75NTR.

Dlx and Msx, which belong to the homeobox gene family, have been widely recognized as key factors in craniofacial and tooth development.^35^, ^36^ Evidence has shown that Msx1 was expressed in the mesenchyme subjacent to the dental lamina of mouse E11.5 d embryos, and its expression became the strongest in the dental papilla and dental follicle at the cap stage (E13.5 d).^37^, ^38^ Dlx1 was also reportedly strongly expressed in the areas near epithelial‐mesenchymal interactions.^39^ Interestingly, we showed that p75NTR and Mage‐D1 were strongly expressed in the mesenchyme of the dental papilla and dental follicle and the inner enamel epithelium, similar to the expression of Dlx1 and Msx1 reported in previous studies. Therefore, we speculated that there might be connections between p75NTR, Mage‐D1 and Dlx/Msx. In this study, Dlx1 and Msx1 were expressed at low levels in p75NTR^ExIII−/−^ EMSCs, implicating their positive correlation with p75NTR. Mage‐D1 was also found to be positively correlated with p75NTR, and thus, we suggest that certain unknown correlations exist between these three factors. It has been verified in previous studies that Mage‐D1 could bind to the intracellular domain of p75NTR via the Mage‐D1 homology domain^40^ and bind to the Dlx/Msx family via the Mage‐D1 interspersed repeat domain.^41^ In other words, Mage‐D1 can bind to both p75NTR and Dlx/Msx. Mage‐D1 was speculated to play a bridging role between the cell membrane receptor p75NTR and the nuclear transcription factors Dlx/Msx. However, further studies are needed to determine the exact mechanism underlying this process.

In summary, our data show that p75NTR is positively correlated with up‐regulation in mineralization of both in vivo p75NTR‐knockout mice and in vitro EMSCs. p75NTR not only promotes osteogenic differentiation and tissue mineralization, but also shows a possible relationship with the circadian rhythm of dental hard tissue formation. p75NTR is positively correlated to Mage‐D1, Dlx1, and Msx1, and Mage‐D1 was speculated to play a bridging role between them. However, further studies are needed to validate how the p75NTR‐Mage‐D1‐Dlx/Msx signalling axis operates during tooth morphogenesis.

## CONFLICT OF INTEREST

All authors declare that they have no competing interests.

## AUTHOR CONTRIBUTIONS

All authors contributed to the study concept and design. ZM and LG carried out the in vivo experiments on p75NTR‐knockout mice. WY, YK and LC carried out the in vitro experiments on ectomesenchymal stem cells. ZM and LJ collected the data. ZM, WX and SJ performed the analysis. ZM, WY, WX and SJ drafted the manuscript. All authors have reviewed and approved the manuscript.

## Data Availability

The data that support the findings of this study are available from the corresponding author upon reasonable request.
